# Partial limitation of cellular functions and compensatory modulation of unfolded protein response pathways caused by double-knockout of ATF6α and ATF6β

**DOI:** 10.1016/j.cstres.2023.11.002

**Published:** 2023-11-20

**Authors:** Ryoko Akai, Hisayo Hamashima, Michiko Saito, Kenji Kohno, Takao Iwawaki

**Affiliations:** 1 Department of Life Science, Medical Research Institute, Kanazawa Medical University, 1-1 Daigaku, Uchinada, Kahoku, Ishikawa 920-0293, Japan; 2 Bio-science Research Center, Kyoto Pharmaceutical University, 1, Misasagishichono-cho, Yamashina-ku, Kyoto 607-8412, Japan; 3 Department of Biochemistry and Molecular Biology, Graduate School of Science, University of Hyogo, Harima Science Garden City, Hyogo 678-1297, Japan; 4 Institute for Research Initiatives, Nara Institute of Science and Technology, 8916-5 Takayama, Ikoma, Nara 630-0192, Japan

**Keywords:** ER stress, BiP, Insulin, Pancreatic β cell

## Abstract

Mammalian cells have three types of endoplasmic reticulum (ER) stress-sensing molecules: ATF6, IRE1, and PERK. Among these, ATF6 is unique in that it is processed in an ER-stress-specific manner and functions as a transcription factor for the activation of anti-ER stress genes (such as BiP). ATF6 is known to have two homologues, ATF6α and ATF6β, and a greater understanding of their functions has been achieved through analyses using cultured cells. Physiological functions are also gradually being investigated in mice lacking ATF6α or ATF6β. However, little is known about the effects on mouse organisms of the deletion of both the ATF6α and ATF6β genes, since such double-knockout (DKO) mice suffer embryonic lethality at an early developmental stage. In this study, we generated and analyzed ATF6 DKO mice in which embryonic lethality was evaded by using Cre/loxP technology. Pancreatic β cell-specific ATF6 DKO mice were born normally and lived without dysregulation of blood-glucose levels but had a reduced tolerance to glucose. Islets isolated from ATF6 DKO mice also showed low production and secretion of insulin and mild enhancement of IRE1 and PERK activity. We further examined the developmental abnormalities of systemic ATF6 DKO mice. The phenotypes of ATF6α^−/−^; ATF6β^−/−^ mice were similar to those previously reported, but ATF6α^+/−^; ATF6β^−/−^ and ATF6α^−/−^; ATF6β^+/−^ mice showed embryonic lethality at middle developmental stages, unlike those reported. Analysis of embryonic fibroblasts derived from these mice revealed that ATF6α and ATF6β have a gene-dose-dependent functional redundancy and display distinct differences in their ability to induce BiP expression. (250 words)

## Introduction

Endoplasmic reticulum (ER) stress has been well studied because of its close association with various biological functions and disease pathogeneses.^15^, ^19^, ^31^ The ER has intrinsic functions related to the synthesis, modification, and transport of secretory and membrane proteins, as well as to membrane lipid homeostasis and calcium storage.^2^ Disturbances in these functions are considered to be ER stresses and are mainly sensed by three ER membrane-localized proteins: ATF6, IRE1, and PERK.^8^, ^11^, ^12^, ^25^, ^30^, ^34^

On sensing an ER stress, ATF6 is transported from the ER to the Golgi apparatus. The transported ATF6 is processed by S1P and S2P in the Golgi apparatus.^33^ A cytoplasmic fragment of 50–60 kDa derived from this processing is translocated to the nucleus.^11^, ^34^ Unlike ATF6, IRE1 is autophosphorylated during ER stress and activates the RNase domain within the same molecule.^12^, ^26^ This RNase domain acts on specific RNA sequences. XBP1 mRNA is one of the substrates recognized by IRE1, and cleavage of XBP1 mRNA by IRE1 induces unique spliceosome-independent splicing.^35^ PERK is also autophosphorylated by ER stress and further functions as a kinase that phosphorylates eIF2α.^8^ Phosphorylated eIF2α attenuates the formation of the translation-initiation complex and promotes ATF4 protein expression.^9^ In addition, nuclear-translocated ATF6, spliced XBP1, and promoted ATF4 all perform as transcription factors to control the expression of genes that reduce ER stress, such as BiP, an ER molecular chaperone.^9^, ^11^, ^12^, ^25^, ^30^, ^34^ These molecular-signaling functions, known as the unfolded protein response (UPR), are evident from studies at the cellular level.

The functions of PERK and IRE1 are becoming better understood through studies with genetically modified animals. Some PERK-deficient mice show striking defects in pancreatic tissue that critically impair the functions to regulate blood-glucose levels and to digest food.^10^ Other PERK-deficient mice display skeletal dysplasia at the developmental stage, resulting in delayed postnatal growth.^36^ In addition, PERK is known to be a gene responsible for Walcott–Rallison syndrome.^5^ In mammals, IRE1 consists of two homologous genes, IRE1α and IRE1β.^12^, ^25^, ^30^ IRE1α-deficient and IRE1β-deficient mice have been generated and analyzed by us and other research groups, and have been shown to have several distinct phenotypes. IRE1α-deficient mice have defects in placental angiogenesis, and they display abnormal liver development, reaching lethality after about 11–12 days of gestation.^13^, ^37^ Furthermore, IRE1α-conditional KO mice have been reported to exhibit pathological features of various diseases, such as diabetes or hepatosteatosis.^14^, ^23^, ^27^, ^38^ IRE1β-deficient mice grow and reproduce normally under normal rearing conditions, but their pathological phenotype is more severe than that of wild-type mice under conditions of intestinal inflammation.^3^ IRE1β has also been shown to play an essential role in optimal mucin production by goblet cells^28^.

The function of ATF6 is also being analyzed by using genetically modified animals, although the research on ATF6 is at an earlier phase compared with that on PERK or IRE1. In mammals, ATF6 also consists of two homologous genes, ATF6α and ATF6β.^11^, ^34^ Both ATF6α-deficient and ATF6β-deficient mice are born and fertile according to the Mendelian rules, but ATF6-double-deficient mice die during early stages of their development (probably before implantation).^32^ Consequently, the effects on biological functions of the loss of both ATF6 genes remain unknown. In this study, we generated and analyzed ATF6-double-deficient mice in which embryonic lethality was prevented by Cre/loxP technology. In particular, we focused on the compensatory activation of the IRE1 and PERK pathways due to ATF6 double deletion and on the similarities and differences in cellular functions (UPR induction and cell proliferation) between ATF6α and ATF6β.

## Materials and methods

### Gene constructs

For ATF6α-conditional KO mice, pKOV2-ATF6αCKO-1 was constructed by the insertion of ATF6α CKO 5′-arm, ATF6α CKO targeting-region, and ATF6α CKO 3′-arm into the *Kpn*I/*Xho*I, *Xho*I/*Hin*dIII, and *Bam*HI/*Eco*RI sites, respectively, of pKOV2. For ATF6β-conditional KO mice, pKOV2-ATF6βCKO-1 was constructed by the insertion of ATF6β CKO 5′-arm, ATF6β CKO targeting region, and ATF6β CKO 3′-arm into the *Kpn*I/*Xho*I, *Xho*I/*Hin*dIII, and *Bam*HI/*Eco*RI sites, respectively, of pKOV2. The ATF6α CKO 5′-arm, ATF6α CKO targeting-region, ATF6α CKO 3′-arm, ATF6β CKO 5′-arm, ATF6β CKO targeting-region, and ATF6β CKO 3′-arm were produced by PCR with mouse genome DNA as a template. The PCR primers are listed in Supplementary Table S1.

### Animals

ATF6α-conditional KO mice and ATF6β-conditional KO mice were generated with ES cells (RF8) as previously described.^17^, ^22^ ROSA26^Flp/Flp^,^7^ RIP-Cre,^21^ and Mox2^+/Cre^^24^ transgenic mice were obtained from the Jackson Laboratory. ROSA26^Flp/Flp^ transgenic mice were used for excision of the Neo gene cassette from the genome of mice recombined with pKOV2-ATF6αCKO-1 or pKOV2-ATF6βCKO-1. RIP-Cre transgenic mice were used for pancreatic β cell-specific deletion of exon 8 from the *ATF6α* allele or exon 10/11 from the *ATF6β* allele. Mox2^+/Cre^ transgenic mice were used for systemic deletion of the preceding exons. All mice were maintained on a mixed (C57BL/6×129/SvE) background, and were fed with CE-2 (CLEA Japan, Inc., Tokyo, Japan) as a normal diet or Quick Fat (CLEA Japan, Inc.) as a high-fat diet. The experimental protocols (#2020–34) that involved animals were approved by the Kanazawa Medical University Institutional Animal Care and Use Committee; all experiments were performed in accordance with the appropriate institutional guidelines.

### Genotyping

**Fig. 1:**
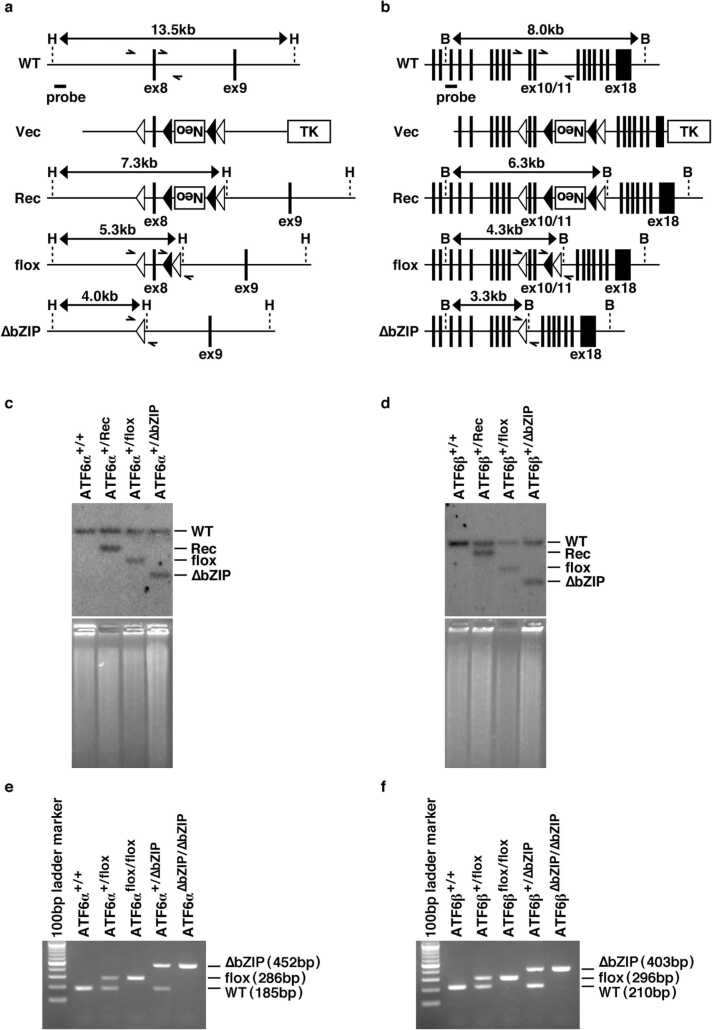
Design and establishment of ATF6α and ATF6β conditional knockout mice. **a, b** Schematic diagrams showing the targeting vector and the indicated genotype in the mouse *ATF6α* and *ATF6β* loci, respectively. “Neo” and “TK” indicate expression units of the neomycin-resistance gene and thymidine kinase gene for positive and negative selection, respectively. Closed and open arrowheads indicate FRT and loxP elements, respectively. WT: wild-type allele, Vec: targeting vector, Rec: homologous recombination allele, flox: flox allele, ∆bZIP: ∆bZIP allele, H: *Hin*dIII site, and B: *Bam*HI. **c, d** Southern blot analysis of mouse genome DNA with the indicated genotype. For genotyping of *ATF6α* and *ATF6β* alleles, mouse genome DNA was digested with *Hin*dIII and *Bam*HI, respectively. The upper panels show autoradiographs with the probes indicated in **a** and **b**, respectively. The lower panels show EtBr staining of genome DNA as a loading control. **e, f** PCR analysis of mouse genome DNA with the indicated genotype. The position of primers is schematically indicated in **a** and **b**, respectively.

For Southern blot analysis of ATF6α-conditional KO mice and ATF6β-conditional KO mice, genome DNA was extracted from mouse tail tips in accordance with the standard procedure. Aliquots (10 μg) of genome DNA were digested with the restriction enzymes shown in Fig. 1a, then loaded into separate lanes of 0.8 % agarose gels and transferred onto nylon membranes (Cat#60207; PALL, Port Washington, NY). Hybridization was performed in H solution (0.5 M Na_2_HPO_4_, 1 mM EDTA, and 7 % SDS) at 65 °C for 16 h. The membranes were then washed four times with W solution (40 mM Na_2_HPO_4_ and 1 % SDS) at 65 °C for 10 min. The ^32^P-labeled DNA fragments derived from ATF6α CKO 5′-pro or ATF6β CKO 5′-pro were used as probes. ATF6α CKO 5′-pro and ATF6β CKO 5′-pro were produced by PCR with mouse genome DNA as a template. The PCR primers are listed in Supplementary Table S1. For PCR analysis of ATF6α-conditional KO mice and ATF6β-conditional KO mice, the relevant specific primer sets that were used are listed in Supplementary Table S2. The genome DNA used for PCR templates was extracted as described above. Genotyping of ROSA26^Flp/Flp^, RIP-Cre, and Mox2^+/Cre^ transgenic mice was performed in accordance with the Jackson Laboratory protocols.

### Measurement of blood glucose and insulin

**Fig. 2:**
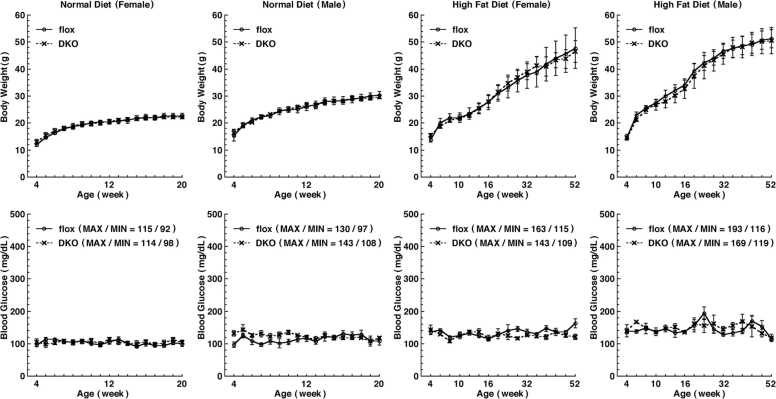
Body weight (upper) and blood glucose (lower) of mice with pancreatic β cell-specific deletion of ATF6α and ATF6β (DKO). Mice with ATF6α^flox/flox^ and ATF6β^flox/flox^ (flox) were used as controls. The plots show the mean, and the error bars denote the S.E.M. (*n* = 5).

**Fig. 3:**
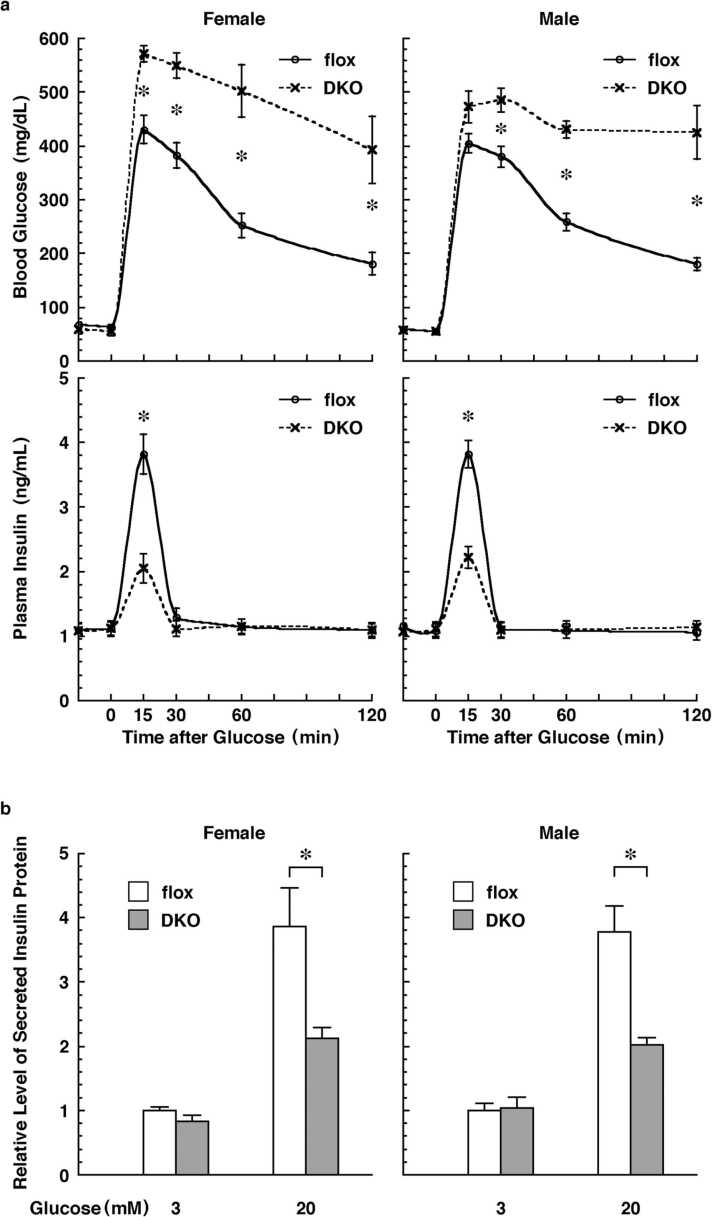
Glucose tolerance of mice and isolated islets with pancreatic β cell-specific deletion of ATF6α and ATF6β (DKO). **a,** Time course of the blood-glucose level (upper) and the blood-insulin level (lower) in mice after oral administration of glucose. The plots show the mean and error bars denote the S.E.M. (*n* = 5). **b**, Relative level of insulin protein secreted from the islets after low- or high-glucose stimulation. All data are normalized to the no-glucose treatment. The columns show the mean, and the error bars denote the S.E.M. (*n* = 3). Mice and the isolated islets with ATF6α^flox/flox^ and ATF6β^flox/flox^ (flox) were used as controls. The asterisks indicate statistically significant results.

The blood-glucose level was measured by using a portable glucose-measuring device (Arkray, Kyoto, Japan). Insulin level was determined by using enzyme-linked immunosorbent assay (ELISA) kits (Cat#AKRIN-031, Shibayagi, Shibukawa, Japan). Glucose-tolerance tests were performed on 11-week-old ATF6 DKO and control mice that had been fasted for 16 h. The mice were then orally administered with 2 mg/g body weight of glucose. Their blood-glucose levels and serum-insulin levels were then measured at the intervals shown in Fig. 2, Fig. 3a.

### Islet isolation and glucose stimulation

Islets were isolated from 14-week-old mice in accordance with a previously reported procedure.^16^ For glucose stimulation of the isolated islets, a pancreatic islet culture kit (Cat#PNI13, Cosmo Bio Co., LTD, Tokyo, Japan) was used in accordance with the manufacturer’s instructions. Incidentally, the analytical conditions (3 and 20 mM glucose) also complied with the same instructions.

### Protein preparation, Western blot analysis, and ELISA

Islets were lysed in Mammal Tissue Extraction Reagent (Cat#AR0101, Boster Biological Technology, Pleasanton, CA). For Western blot analysis, the lysate was heated in an SDS sample buffer [50 mM Tris-HCl (pH 6.8), 2 % SDS, 50 mM DTT, 10 % glycerol, and 1 mg/mL Bromophenol Blue] at 98 °C for 10 min. SDS-PAGE was then performed to resolve the proteins in the lysate. After electrophoresis, the proteins were electrotransferred onto a poly(vinylidene fluoride) microporous membrane and immunodetected with anti-insulin polyclonal antibody (Cat#A0564, DAKO, Glostrup, Denmark) and anti-GAPDH monoclonal antibody (Cat#2118, Cell Signaling Technology, Beverly, MA). The total protein levels in the samples subjected to SDS-PAGE were confirmed by using a gel stain reagent (Cat#161–0495, Bio-Rad Laboratories, Hercules, CA). For ELISA of ER-stress-responsive molecules, proteins in the lysate were also coated onto multi-well plates and immunodetected with the appropriate antibodies in accordance with the manufacturer’s protocol (https://www.ptglab.com/media/1604/protocols-for-web_indirect-elisa.pdf). The following antibodies were used for ELISA: anti-GAPDH monoclonal antibody (Cat#2118, Cell Signaling Technology), anti-ATF4 polyclonal antibody (Cat#11815, Cell Signaling Technology), anti-eIF2α monoclonal antibody (Cat#5324, Cell Signaling Technology), anti-phospho-eIF2α (Ser51) monoclonal antibody (Cat#3398, Cell Signaling Technology), anti-PERK monoclonal antibody (Cat#3192, Cell Signaling Technology), anti-phospho-PERK (Thr980) monoclonal antibody (Cat#3179, Cell Signaling Technology), anti-IRE1α monoclonal antibody (Cat#3294, Cell Signaling Technology), and our self-produced anti-phospho-IRE1α (Ser724) polyclonal antibody^1^. Total and mature insulin protein in the lysate was quantified by using commercial ELISA kits (Cat#AKRIN-031 and Cat#AKRIN-011S, Shibayagi) in accordance with the manufacturer’s instructions. The total protein level in the lysate was determined by using a commercial BCA assay kit (Cat#23227, Pierce Biotechnology, Rockford, IL) and the result was used as an internal control in the analysis of insulin and ATF4. All results were expressed as the mean ± SEM of triplicate experiments performed with islets derived from three mice.

### RNA preparation and quantitative PCR

Total RNA was extracted from the islets or mouse embryonic fibroblasts (MEFs) by using the Isogen reagent (Cat#311–02501, Nippon Gene, Tokyo, Japan). A SuperScript first-strand synthesis system (Cat#11904–018, Life Technologies, Carlsbad, CA) was used to synthesize the cDNA in accordance with the manufacturer’s instructions. Quantitative PCR analysis of the various transcripts was performed by using TaqMan probe and QuantStudio 12 K Flex (Applied Biosystems, Waltham, MA) in accordance with the manufacturer’s instructions. The GAPDH transcript was used as an internal control in each analysis. The results of the quantitative PCR analyses are reported as the mean ± SEM from triplicate experiments performed with islets derived from three mice or three MEF cultures. Probe/primer sets Mm01259683_g1, Mm00731595_gH, Mm01295328_m1, Mm00444369_g1, Mm00517691_m1, Mm00551747_m1, Mm00435119_m1, and Mm99999915_g1 (Applied Biosystems) were used for quantification of Ins1, Ins2, ATF6α, ATF6β, BiP, CreP, GADD34, and GAPDH transcripts, respectively. Mouse total XBP1 transcripts were quantified by using the forward primer 5′-gaa tgg aca cgc tgg atc ct-3′, the reverse primer 5′-gcc acc agc ctt act cca ctc-3′, and the probe 5′-FAM-cct ctg gaa cct cg-MGB-3′. Mouse spliced XBP1 transcripts were quantified by using the forward primer 5′-gaa tgg aca cgc tgg atc ct-3′, the reverse primer 5′-cag agt cca tgg gaa gat gtt ct-3′, and the probe 5′-FAM-cac ctg ctg cgg act-MGB-3′.

### Cell culture

MEFs were collected from each mouse embryo at 11.5 days *post coitum* in accordance with a previously described procedure^18^ and then cultured in Dulbecco’s modified Eagle’s medium supplemented with 10 % fetal bovine serum at 37 °C under 5 % CO_2_. The cell number was determined by using a Burker–Turk hemocytometer (Cat#03–303–1, Erma, Yoshikawa, Japan). To induce an ER stress response, cells were treated with 15 μg/mL of tunicamycin (Cat#T7765; Sigma-Aldrich, Saint Louis, MO), 3 μM of thapsigargin (Cat#33637–31; nacalai tesque, Kyoto, Japan), or 0.3 mM of dithiothreitol (Cat#14112–94; nacalai tesque) for 6 h.

### Microscopy

Embryos were observed by using a stereoscopic microscope (Cat#MVX10, Olympus, Tokyo, Japan) equipped with a color charge-coupled device camera (Cat#DP72, Olympus). MEFs were observed with a phase-contrast microscope (Cat#BZ-X810, Keyence, Osaka, Japan).

### Statistical analysis

Statistical analyses were performed by using *KaleidaGraph*, version 4.1, software (Synergy Software, Reading, PA). The differences indicated in each figure were analyzed by using Student’s *t*-test: a probability of 0.05 was considered to be significant.

## Results

### Generation of mice with Cre/loxP-dependent deletion of ATF6α and ATF6β

To ensure the loss of both ATF6α and ATF6β functions, we considered it a reasonable strategy to exclude the DNA-binding region, which is important as a transcription factor, from the genome. In *ATF6α* and *ATF6β*, exons 8 and 10, respectively, are known to encode the DNA-binding region from the genome database.^11^ We therefore designed a plan that permitted Cre/loxP-dependent deletion of those exons (encoding the DNA-binding region) for both *ATF6α* and *ATF6β* genes (Fig. 1a and b). We then introduced the targeting vectors for both *ATF6α* and *ATF6β* genes into ES cells to obtain homologous recombinants. We further established mouse lines (ATF6α^+/Rec^ and ATF6β^+/Rec^) derived from the corresponding homologous recombinational ES cells by using mouse embryological engineering. By Southern blot analysis, we confirmed that mating these mouse lines with Flp- and Cre-expressing mice yielded ATF6α^+/flox^, ATF6α^+/∆bZIP^, ATF6β^+/flox^, and ATF6β^+/∆bZIP^ mouse lines as expected (Fig. 1c and d). In addition, the genotypes of mice born in each F2 generation could be distinguished by means of PCR using specific primers (Fig. 1e and f).

### Characterization of mice with pancreatic β cell-specific deletion of ATF6α and/or ATF6β

It has been reported that ER stress is induced and ATF6 is constitutively activated in insulin-producing cells.^1^ We therefore hypothesized that ATF6 is essential for pancreatic β cell function. To examine this hypothesis, changes in blood-glucose levels and in body weight were measured every week or every other week under normal or high-fat diets, respectively (Fig. 2). Regardless of the diet type, DKO mice with deletions of ATF6α and ATF6β in their pancreatic β cells specifically (produced by mating with RIP-Cre mice), had blood-glucose levels comparable to those of control ATF6α^flox/flox^; ATF6β^flox/flox^ mice (flox mice) at young and old stages. Also, there was no marked difference in body weight between the DKO and the flox mice.

Next, to apply a strong load to their pancreatic β cells, the mice were orally administered with a high concentration of glucose in water. Blood-glucose levels of DKO mice were significantly higher than those of flox mice at 15–120 min after administration. Plasma insulin levels of DKO mice were significantly lower than those of flox mice at 15 min after administration (Fig. 3a).

To compare insulin secretion by pancreatic β cells, islets isolated from DKO and flox mice were cultured in low-glucose (3 mM) or high-glucose (20 mM) media, respectively. Under low-glucose culture conditions, insulin levels in the culture medium of DKO islets were comparable to those of flox islets. On the other hand, the insulin levels in the culture medium of flox islets under high-glucose culture conditions were four times higher than those under low-glucose culture conditions, whereas the insulin levels in the culture medium of DKO islets under high-glucose culture conditions were only two times higher than those under low-glucose culture conditions (Fig. 3b).

**Fig. 4:**
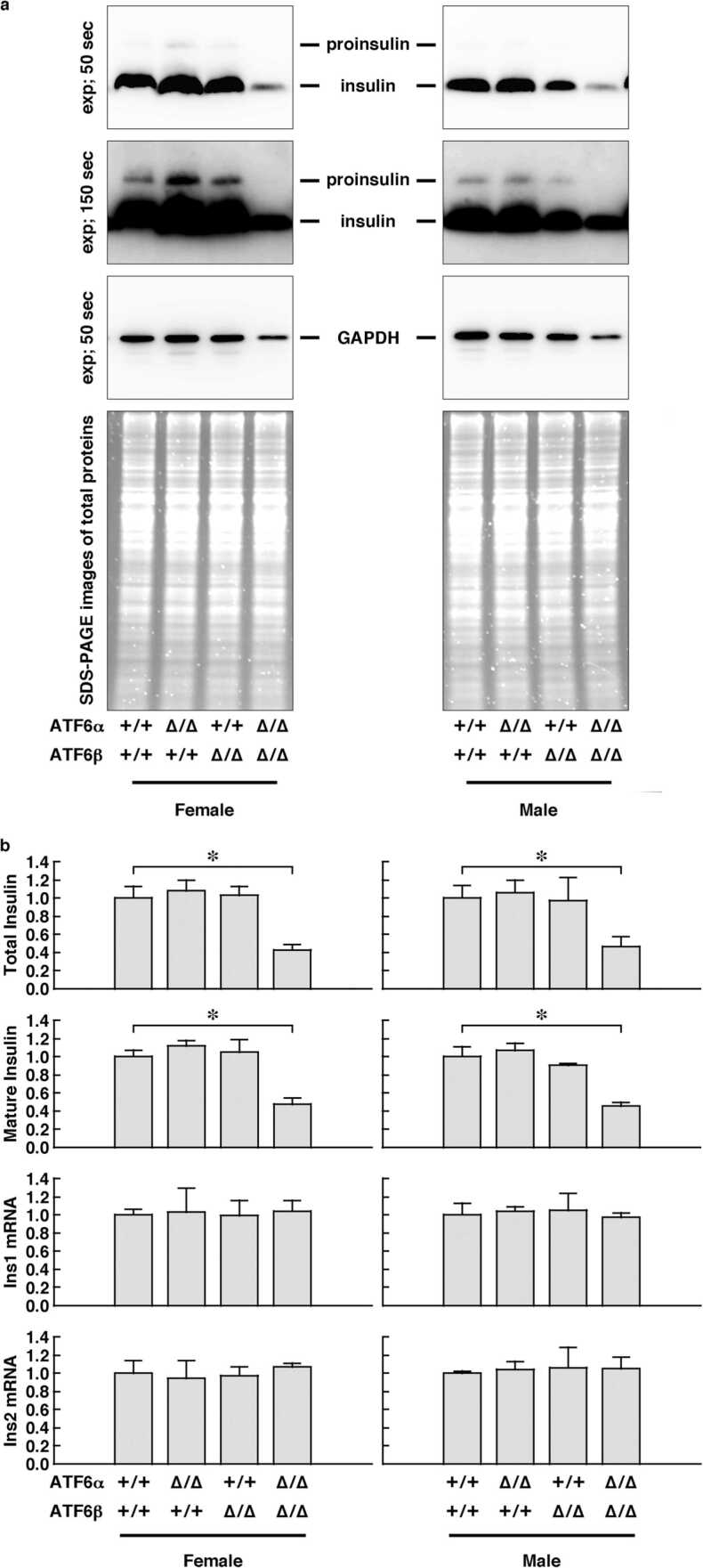
Expression levels of insulin protein and mRNA in islets isolated from wild-type or ATF6-mutant mice. **a,** Each panel shows the Western blot signals as detected by anti-insulin antibody or anti-GAPDH antibody. Signals of GAPDH and images from SDS-PAGE were used as a loading control. **b**, Expression levels of insulin protein and mRNA as measured by ELISA and quantitative PCR analysis, respectively. The columns show the mean, and the error bars denote the S.E.M. (*n* = 3). The asterisks indicate statistically significant results. + = wild-type allele; ∆ = ∆bZIP allele.

To compare expression levels of insulin protein in pancreatic β cells, Western blot and ELISA analyses using anti-insulin antibodies were performed on lysates of islets isolated from mice. The expression levels of insulin protein in ATF6α^∆bZIP/∆bZIP^; ATF6β^+/+^ (ATF6α KO) mice and ATF6α^+/+^; ATF6β^∆bZIP/∆bZIP^ (ATF6β KO) mice were comparable to those in ATF6α^+/+^; ATF6β^+/+^ (wild-type) mice, whereas those of DKO mice were significantly decreased. However, quantitative PCR analysis showed that expression levels of insulin mRNA in DKO mice were comparable to those in the other three groups (Fig. 4). In Fig. 4b, the insulin protein levels are normalized at the total protein levels, whereas in Supplementary Fig. S1, they are collaterally normalized at the GAPDH protein levels and insulin mRNA levels.

**Fig. 5:**
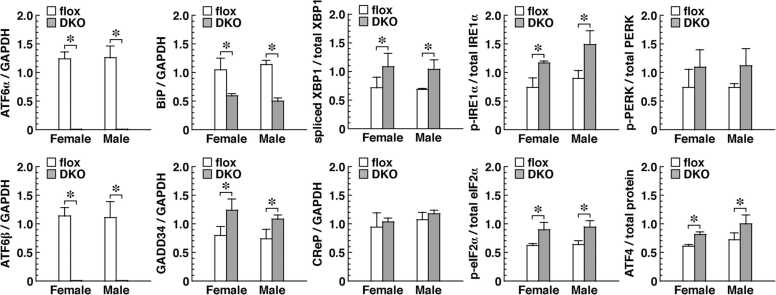
Expression levels or phosphorylation levels of ER-stress-associated molecules in isolated islets with pancreatic β cell-specific deletion of ATF6α and ATF6β (DKO). IRE1α, PERK, and eIF2α were evaluated from the phosphorylation level of the protein. ATF4 was evaluated from the expression level of the protein. The other values were evaluated from the expression level of mRNA. Islets with ATF6α^flox/flox^ and ATF6β^flox/flox^ (flox) were used as a control. The columns show the mean, and the error bars denote the S.E.M. (*n* = 3). The asterisks indicate statistically significant results.

To assess the impact of ATF6-double-deficiency in pancreatic β cells, we compared the activation levels of ER-stress-responsive molecules between islets isolated from flox and DKO mice. We confirmed that the expression of ATF6α mRNA and ATF6β mRNA was barely detectable in DKO mice, as expected. These analyses also revealed that the expression level of BiP mRNA in DKO mice was approximately half that in flox mice. On the other hand, the phosphorylation levels of IRE1α and PERK proteins were 30–50 % higher in DKO mice. The levels of XBP1 splicing, eIF2α phosphorylation, and ATF4 protein expression were also consistently 20–30 % higher in DKO mice than in flox mice. In addition, we examined the expression levels of two molecules, GADD34 and CReP, that regulate eIF2α dephosphorylation. Expression levels of GADD34 mRNA, which is transcriptionally induced by ATF4, were 30–40 % higher in DKO mice than in flox mice, whereas expression levels of the CReP mRNA, which is not transcriptionally induced by ATF4, in DKO mice were comparable with those in flox mice (Fig. 5). The data shown in Fig. 5 were also consistent with the results of Western blot analyses and reverse transcription PCR (RT-PCR) analyses (Supplementary Figs. S2 and S3). These results applied to both females and males (Fig. 2, Fig. 3, Fig. 4, Fig. 5 and Supplementary Figs. S1–S3).

### Characterization of mouse embryos with systemic deletion of ATF6α and/or ATF6β

**Fig. 6:**
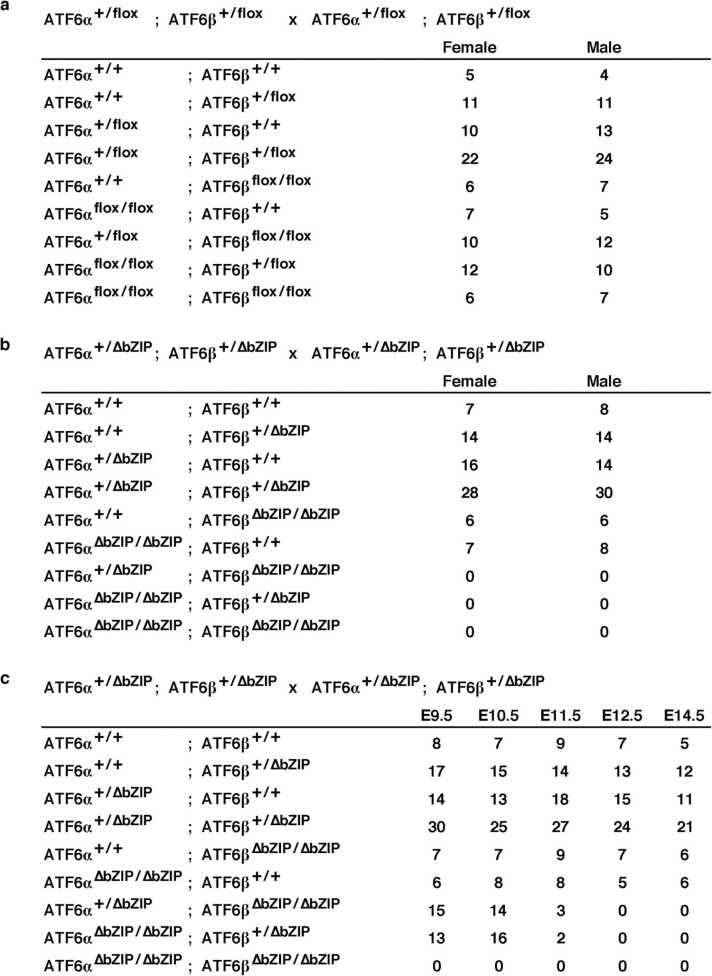
Numbers of living offspring obtained from matings between ATF6-mutant mice. **a,** Number of living pups obtained from matings between ATF6α^+/flox^; ATF6β^+/flox^ mice. **b,** Number of living pups obtained from matings between ATF6α^+/∆bZIP^; ATF6β^+/∆bZIP^ mice. **c,** Number of living embryos obtained from matings between ATF6α^+/∆bZIP^; ATF6β^+/∆bZIP^ mice. The viability of the embryos was judged from the beating of their hearts.

Conventional ATF6-double-deficient mice have previously been reported to exhibit embryonic lethality. Systemic deletion of ATF6α and ATF6β (ATF6α^∆bZIP/∆bZIP^; ATF6β^∆bZIP/∆bZIP^) can be also analyzed by mating between *Mox2*^*+/Cre*^ transgenic mice and ATF6α^flox/flox^; ATF6β^flox/flox^ mice. To confirm that our ATF6α^∆bZIP/∆bZIP^; ATF6β^∆bZIP/∆bZIP^ mice were embryonically lethal and that ATF6α^flox/flox^; ATF6β^flox/flox^ mice were born normally, we mated female and male ATF6α^+/∆bZIP^; ATF6β^+/∆bZIP^ mice, and female and male ATF6α^+/flox^; ATF6β^+/flox^ mice. From the latter mating, mice of all putative genotypes were born according to Mendelian rules, and these developed normally (Fig. 6a). The former mating did not give rise to ATF6α^∆bZIP/∆bZIP^; ATF6β^+/∆bZIP^ mice, ATF6α^+/∆bZIP^; ATF6β^∆bZIP/∆bZIP^ mice, and ATF6α^∆bZIP/∆bZIP^; ATF6β^∆bZIP/∆bZIP^ mice, but the other mouse genotypes were born according to Mendelian rules (Fig. 6b).

**Fig. 7:**
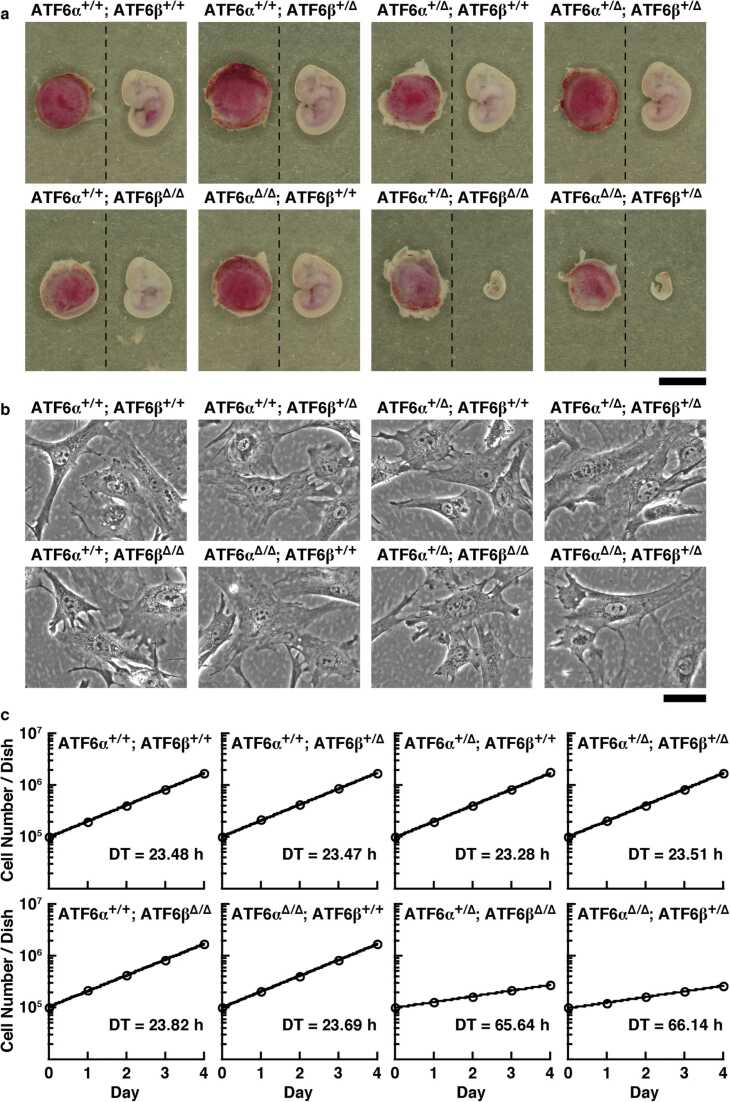
Characterization of embryos and MEFs with ATF6α^+/∆bZIP^; ATF6β^∆bZIP/∆bZIP^ or ATF6α^∆bZIP/∆bZIP^; ATF6β^+/∆bZIP^. **a,** Typical images of embryos and placentas with the indicated ATF6 genotype at E11.5 stage (scale bar: 5 mm). **b,** Typical images of MEFs with the indicated ATF6 genotype (scale bar: 50 µm). **c,** Typical growth curves for MEFs with the indicated ATF6 genotype (DT: doubling time calculated from the growth curve). The MEFs with ATF6α^+/+^; ATF6β^+/+^ (wild-type) were used as a control. + = wild-type allele, and ∆ = ∆bZIP allele.

To address the developmental phenotype of the offspring from mating of ATF6α^+/∆bZIP^; ATF6β^+/∆bZIP^ mice, we next compared the viability and appearance among all genotypes of offspring at the embryonic stages. Living ATF6α^∆bZIP/∆bZIP^; ATF6β^∆bZIP/∆bZIP^ mice were not obtained at all in the five embryonic stages examined. Living ATF6α^∆bZIP/∆bZIP^; ATF6β^+/∆bZIP^ mice and ATF6α^+/∆bZIP^; ATF6β^∆bZIP/∆bZIP^ mice were obtained in stages before embryonic day 11.5 (E11.5), but not after E12.5 (Fig. 6c). ATF6α^∆bZIP/∆bZIP^; ATF6β^+/∆bZIP^ embryos and ATF6α^+/∆bZIP^; ATF6β^∆bZIP/∆bZIP^ embryos at E11.5 were also much smaller compared with the embryos of other genotypes and showed developmental retardation (Fig. 7a).

### Comparison of responsivity to ER stress among mouse embryonic fibroblasts with various ATF6 genotypes

Previous studies have reported that ATF6α^−/−^; ATF6β^+/−^ and ATF6α^+/−^; ATF6β^−/−^ mice are born alive.^32^ However, no ATF6α^∆bZIP/∆bZIP^; ATF6β^+/∆bZIP^ mice and no ATF6α^+/∆bZIP^; ATF6β^∆bZIP/∆bZIP^ mice were born in this study. We analyzed these mutation-dependent abnormalities by using MEFs, as we were, fortunately, able to obtain MEFs derived from ATF6α^∆bZIP/∆bZIP^; ATF6β^+/∆bZIP^ embryos and ATF6α^+/∆bZIP^; ATF6β^∆bZIP/∆bZIP^ embryos. Comparison of the morphologies of ATF6α^∆bZIP/∆bZIP^; ATF6β^+/∆bZIP^ MEFs and ATF6α^+/∆bZIP^; ATF6β^∆bZIP/∆bZIP^ MEFs in phase-contrast images with those of other genotypes of MEFs did not reveal any significant differences (Fig. 7b). On the other hand, ATF6α^∆bZIP/∆bZIP^; ATF6β^+/∆bZIP^ MEFs and ATF6α^+/∆bZIP^; ATF6β^∆bZIP/∆bZIP^ MEFs proliferated more slowly than cells of other genotypes of MEFs; The difference in doubling time was a nearly threefold (Fig. 7c).

**Fig. 8:**
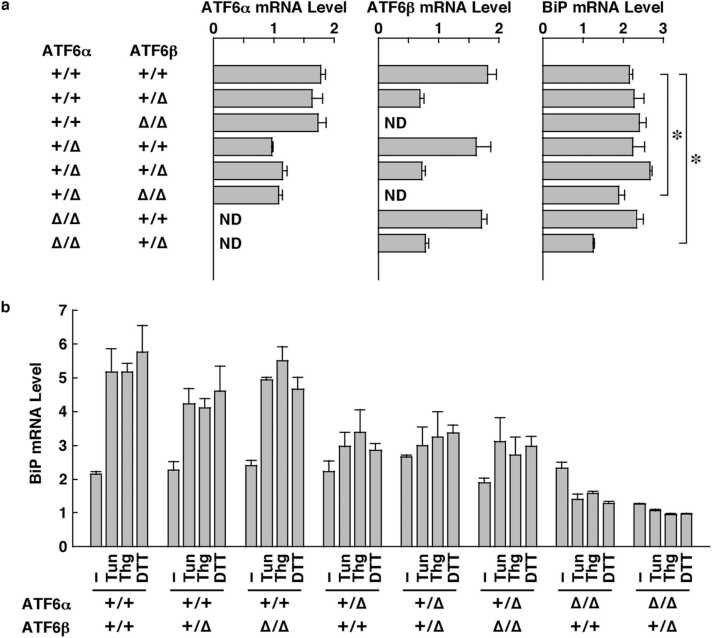
Comparison of ATF6 levels and BiP levels between various ATF6 mutant MEFs. **a,** Expression levels of ATF6α mRNA, ATF6β mRNA, and BiP mRNA in ATF6 mutant MEFs cultured under normal conditions. MEFs with ATF6α^+/+^ and ATF6β^+/+^ (wild-type) were used as controls. The asterisks indicate statistically significant results. **b,** Expression levels of BiP mRNA in ATF6 mutant MEFs cultured under ER stress conditions (Tun, Thg, and DTT). Normal conditions were used as a control. + = wild-type allele, ∆ = ∆bZIP allele, Tun: tunicamycin, Thg: Thapsigargin, DTT: dithiothreitol. The columns indicate the mean, and the error bars denote the S.E.M. (*n* = 3).

The mRNA expression levels of ATF6α and ATF6β in these MEFs were gene-dose dependent. In other words, the expression levels of ATF6α mRNA and ATF6β mRNA in ATF6α^+/∆bZIP^ MEFs and ATF6β^+/∆bZIP^ MEFs were about half of those in ATF6α^+/+^ MEFs and ATF6β^+/+^ MEFs; moreover, ATF6α mRNA and ATF6β mRNA were not detected in ATF6α^∆bZIP/∆bZIP^ MEFs and ATF6β^∆bZIP/∆bZIP^ MEFs, respectively. Expression levels of BiP mRNA were also decreased to 90 % and 60 % in ATF6α^+/∆bZIP^; ATF6β^∆bZIP/∆bZIP^ MEFs and ATF6α^∆bZIP/∆bZIP^; ATF6β^+/∆bZIP^ MEFs, respectively, compared with those of wild-type MEFs (Fig. 8a). Transcription of BiP genes is strongly activated by chemical ER stress. In fact, expression levels of BiP mRNA were increased 2.5-fold by tunicamycin, thapsigargin, or dithiothreitol in wild-type MEFs. Interestingly, the induction of BiP expression by chemical ER stressors in ATF6α^+/+^; ATF6β^+/∆bZIP^ MEFs and ATF6α^+/+^; ATF6β^∆bZIP/∆bZIP^ MEFs was comparable to that in wild-type MEFs, whereas that in ATF6α^+/∆bZIP^; ATF6β^+/+^ MEFs, ATF6α^+/∆bZIP^; ATF6β^+/∆bZIP^ MEFs and ATF6α^+/∆bZIP^; ATF6β^∆bZIP/∆bZIP^ MEFs was markedly weaker than that in wild-type MEFs. In ATF6α^∆bZIP/∆bZIP^; ATF6β^+/+^ MEFs and ATF6α^∆bZIP/∆bZIP^; ATF6β^+/∆bZIP^ MEFs, BiP expression was not induced by chemical ER stressors, and was rather slightly reduced by those stressors (Fig. 8b).

## Discussion

First, we generated a model in which both *ATF6α* and *ATF6β* genes could be deleted in a Cre recombinase-dependent manner, permitting ATF6 DKO mice to be analyzed without embryonic lethality. We were then able to confirm, by Southern blot analysis and genomic PCR analysis, that the mouse models functioned as designed (Fig. 1). Pancreatic β cell-specific double deletion of ATF6 in these mouse models did not cause significant abnormalities in body weight or blood-glucose during the periods examined under ad libitum conditions on normal or high-fat diets (Fig. 2). However, pancreatic β cell-specific ATF6 DKO mice had significantly higher blood-glucose levels and lower plasma-insulin levels than control mice in an oral-glucose-tolerance test (Fig. 3a). Analysis of isolated islets also revealed that expression levels of insulin mRNA were comparable between pancreatic β cell-specific ATF6 DKO and controls, but that expression levels and secretion levels of insulin protein were considerably lower in ATF6 DKO than in controls (Figs. 3b and 4). In addition, analysis of the same islets showed that expression levels of BiP mRNA were decreased in ATF6 DKO, but that activation levels of other ER-stress-response molecules (IRE1α pathway and PERK pathway) increased in ATF6 DKO (Fig. 5). Conventional ATF6 DKO (ATF6α^−/−^; ATF6β^−/−^) mice were reported to have embryonic lethality much earlier in the developmental stage, so we gained the impression that pancreatic β cell-specific ATF6 DKO mice exhibit a milder phenotype than expected. However, our ATF6α^∆bZIP/∆bZIP^; ATF6β^∆bZIP/∆bZIP^ mice had embryonic lethality similar to that of ATF6α^−/−^; ATF6β^−/−^ mice (Fig. 6). Therefore, we do not consider that our gene-targeting strategy had any problems. On the other hand, other genotypes (ATF6α^+/∆bZIP^; ATF6β^∆bZIP/∆bZIP^ and ATF6α^∆bZIP/∆bZIP^; ATF6β^+/∆bZIP^) of mice had developmental retardation and embryonic lethality, unlike those previously reported (Figs. 6 and 7a). We do not know what is responsible for this controversial result, but an analysis using these MEFs revealed that the gene dose of ATF6 dramatically affects the mitotic rate and the expression levels of BiP mRNA in cultured cells (Figs. 7c and 8).

On exposure to high concentrations of glucose, the pancreatic β cells activate acute insulin secretion. In preparation for such conditions, normal pancreatic β cells produce high levels of insulin protein. However, expression levels of insulin protein were lower in ATF6-deficient pancreatic β cells than in ATF6-intact pancreatic β cells. This feature must have diminished the increase in insulin secretion and recovery of blood-glucose levels in glucose-stimulated ATF6 DKO mice.

In the meantime, why were expression levels of insulin protein low in pancreatic β cells of ATF6 DKO mice? In pancreatic β cells of ATF6 DKO mice, IRE1α and PERK were activated in a compensatory manner, and the expression level of spliced XBP1 and the phosphorylation level of eIF2α were increased by IRE1α and PERK respectively (Fig. 5). Because the IRE1α-XBP1 pathway has been shown to activate ER-associated protein degradation^20^ and an ATF6 DKO-dependent ER chaperone deficiency might prevent proinsulin maturation, we speculate that the degradation mechanisms accelerated the quantitative decrease of unfolded and immature proinsulin. Additionally, phosphorylation of eIF2α has been found to repress global translation,^4^ from which finding we speculate that translational repression reduced the protein production of insulin in ATF6 DKO mice. Incidentally, it has been reported that changes in levels of blood glucose and plasma insulin caused by glucose stimulation of mice lacking ATF6α alone are comparable with those of wild-type mice,^29^ and that those of mice lacking ATF6β alone are only slightly different from those of control mice^6^.

Next, why was the effect of ATF6 DKO less pronounced under ad libitum conditions on normal or high-fat diets? Compensatory activation of IRE1α and PERK in pancreatic β cells of ATF6 DKO is only partial, which does not severely decrease insulin levels under those conditions. In other words, the expression levels of insulin protein in pancreatic β cells of ATF6 DKO mice might have been capable of adapting to elevated blood-glucose levels under ad libitum conditions on normal or high-fat diets in a similar manner to those of the control mice.

ATF6α^∆bZIP/∆bZIP^; ATF6β^∆bZIP/∆bZIP^ mice displayed embryonic lethality at early developmental stages, whereas ATF6α^∆bZIP/∆bZIP^; ATF6β^+/+^ mice and ATF6α^+/+^; ATF6β^∆bZIP/∆bZIP^ mice were viable. ATF6α^+/∆bZIP^; ATF6β^∆bZIP/∆bZIP^ mice and ATF6α^∆bZIP/∆bZIP^; ATF6β^+/∆bZIP^ mice also showed a common phenotype. These results suggest that ATF6α and ATF6β have redundant functions. However, different properties of ATF6α and ATF6β were also revealed from an expression analysis of BiP mRNA. Regardless of the ATF6β genotype, deletion of ATF6α resulted in complete inhibition of ER-stress-dependent transcriptional induction of the *BiP* gene, which was partially diminished when ATF6α was heterozygous. These results indicate that ER stress-dependent induction of BiP mRNA is regulated by ATF6α, but not by ATF6β. On the other hand, Fig. 8a shows that both ATF6α and ATF6β independently contribute to the basal expression level of BiP mRNA.

In this study, we generated model mice with conditional deletion of both ATF6α and ATF6β. By mating these with various Cre mice, the resulting mice will allow us to analyze the function of both ATF6α and ATF6β at specific ages and in specific tissues/cell types on demand, which had been previously difficult due to the functional redundancy of ATF6α and ATF6β and the lethality of ATF6 double-deficient mice. Analysis of the new mice might reveal novel aspects of the roles played by ATF6α and ATF6β in vivo. From this study, we arrived at the following two conclusions: (1) double deletion of ATF6α and ATF6β in pancreatic β cells causes compensatory activation of other ER-stress-responsive molecules to decrease the expression level of insulin protein, and (2) the partial functional redundancy between ATF6α and ATF6β is gene-dose dependent.

## Author contributions

T.I contributed to the study design, the in vitro research data, and the preparation of the manuscript. R.A. and H. H. contributed to the in vivo research data. M.S. and K.K. contributed to the generation of ATF6α and ATF6β conditional KO mice. T.I. is the guarantor of this work and, as such, had full access to all data in the study and takes responsibility for the integrity of the data and the accuracy of the data analysis.

## Funding

This work was supported by JSPS KAKENHI #24390049 (to T.I.) and #24228002 (to K.K.), and by grants from Kanazawa Medical University (to T.I.), (to T.I.), The Uehara Memorial Foundation (to T.I.), Toray Science Foundationand the Takeda Science Foundation (to K.K.).

## Declaration of Competing Interest

The authors declare the following financial interests/personal relationships which may be considered as potential competing interests: Takao Iwawaki reports financial support was provided by JSPS. Kenji Kohno reports financial support was provided by JSPS. Takao Iwawaki reports financial support was provided by The Uehara Memorial Foundation. Takao Iwawaki reports financial support was provided by Toray Science Foundation. Kenji Kohno reports financial support was provided by The Takeda Science Foundation. If there are other authors, they declare that they have no known competing financial interests or personal relationships that could have appeared to influence the work reported in this paper.

## Data Availability

Data will be made available on request.
