# Supplementary material for: Partial limitation of cellular functions and compensatory modulation of unfolded protein response pathways caused by double-knockout of ATF6α and ATF6β
Source: Cell Stress Chaperones. 2023 Nov 20;29(1):34–48. doi: 10.1016/j.cstres.2023.11.002 (PMC10939067; doi:10.1016/j.cstres.2023.11.002)
Supplement: Supplementary file 1 — Supplementary material [file mmc1.docx]

**Supplementary materials and methods**

RT-PCR

Total RNA was extracted from the islets by using the Isogen reagent (Cat#311-02501, Nippon Gene, Tokyo, Japan). A SuperScript first-strand synthesis system (Cat#11904-018, Life Technologies, Carlsbad, CA) was used to synthesize the cDNA in accordance with the manufacturer’s instructions. ATF6α, ATF6β, BiP, XBP1, GADD34, CReP, and GAPDH cDNAs were amplified by 25 or 30 cycles of PCR with each specific primer set. The PCR primers are listed in Supplementary Table S3. The PCR products subjected to PAGE were detected by staining with EtBr.

Western blot analysis

Islets were lysed in Mammal Tissue Extraction Reagent (Cat#AR0101, Boster Biological Technology, Pleasanton, CA). The lysate was heated in an SDS sample buffer [50 mM Tris-HCl (pH 6.8), 2% SDS, 50 mM DTT, 10% glycerol, and 1 mg/mL Bromophenol Blue] at 98 °C for 10 min. SDS-PAGE was then performed to resolve the proteins in the lysate. After electrophoresis, the proteins were electrotransferred onto a poly(vinylidene fluoride) microporous membrane and immunodetected with anti-ATF4 polyclonal antibody (Cat#11815, Cell Signaling Technology, Beverly, MA), anti-eIF2α monoclonal antibody (Cat#5324, Cell Signaling Technology), anti-phospho-eIF2α (Ser51) monoclonal antibody (Cat#3398, Cell Signaling Technology), anti-PERK monoclonal antibody (Cat#3192, Cell Signaling Technology), anti-phospho-PERK (Thr980) monoclonal antibody (Cat#3179, Cell Signaling Technology), anti-IRE1α monoclonal antibody (Cat#3294, Cell Signaling Technology), and our self-produced anti-phospho-IRE1α (Ser724) polyclonal antibody (Akai et al. 2015). The total protein levels subjected to SDS-PAGE were confirmed with the gel stain reagent (Cat#161-0495, Bio-Rad Laboratories, Hercules, CA).

**References**

Akai R, Hosoda A, Yoshino M, Iwawaki T (2015) Constitutive role of GADD34 and CReP in cancellation of phospho-eIF2α-dependent translational attenuation and insulin biosynthesis in pancreatic β cells. Genes Cells 20:871–886. https://doi.org/10.1111/gtc.12279

**Supplementary figure legends**

Fig. S1

Expression levels of insulin protein as measured by ELISA. **a,** Normalization by GAPDH protein levels. **b,** Normalization by Ins1 mRNA levels. **c,** Normalization by Ins2 mRNA levels. The columns show the mean, and the error bars denote the S.E.M. (*n* = 3). The asterisks indicate statistically significant results. + = wild-type allele; ∆ = ∆bZIP allele.

Fig. S2

RT-PCR signals of ER-stress-associated molecules in isolated islets with pancreatic β cell-specific deletion of ATF6α and ATF6β (DKO). Islets with ATF6α^flox/flox^ and ATF6β^flox/flox^ (flox) were used as a control. Signals of GAPDH were used as a loading control. Three samples were analyzed for each female (F) and male (M). XBP1u = unspliced XBP1; XBP1s = spliced XBP1.

Fig. S3

Western blot signals of ER-stress-associated molecules in isolated islets with pancreatic β cell-specific deletion of ATF6α and ATF6β (DKO). Islets with ATF6α^flox/flox^ and ATF6β^flox/flox^ (flox) were used as a control. Images of SDS-PAGE were used as a loading control. Three samples were analyzed for each female (F) and male (M).
