# Supplementary material for: Partial limitation of cellular functions and compensatory modulation of unfolded protein response pathways caused by double-knockout of ATF6α and ATF6β
Source: Cell Stress Chaperones. 2023 Nov 20;29(1):34–48. doi: 10.1016/j.cstres.2023.11.002 (PMC10939067; doi:10.1016/j.cstres.2023.11.002)
Supplement: Supplementary file 2 — Supplementary material [file mmc2.docx]

|  | **Table S1. Akai R. et al.** |
| --- | --- |
| Table S1. Information on primers for the construction of targeting vectors and probe cloning vectors for Southern blot analysis | |
| Application | Sequence |
| 5' primer for ATF6α CKO 5'-arm | 5'-cggggtaccgtgttccgtggacttcggcacg-3' |
| 3' primer for ATF6α CKO 5'-arm | 5'-ccgctcgagtcccaccccaccccaaatgtttacaagg-3' |
| 5' primer for ATF6α CKO targeting-region | 5'-ccgctcgagataacttcgtatagcatacattatacgaagttatggcagatgcttttccttggcttgg-3' |
| 3' primer for ATF6α CKO targeting-region | 5'-cccaagcttaaccaaccaggtctacacagtgaaacc-3' |
| 5' primer for ATF6α CKO 3'-arm | 5'-cgcggatccaagctttgggacttacatgtacaccggactgaacttgaagttgtgtgg-3' |
| 3' primer for ATF6α CKO 3'-arm | 5'-ccggaattcggacttgggactttgagcctctgg-3' |
| 5' primer for ATF6β CKO 5'-arm | 5'-cggggtaccagatcttcaggtgaagtccgagccatcctctcc-3' |
| 3' primer for ATF6β CKO 5'-arm | 5'-ccgctcgagaacgggctacagctaagaacagttgacttgccttcccactgg-3' |
| 5' primer for ATF6β CKO targeting-region | 5'-ccgctcgagataacttcgtatagcatacattatacgaagttataacttagggctcggaacaagagc-3' |
| 3' primer for ATF6β CKO targeting-region | 5'-cccaagcttttatttgacactgtctctcagcagcctgccattcatcaagg-3' |
| 5' primer for ATF6β CKO 3'-arm | 5'-cgcggatccgagggtgggtcgtgctgagcagtgacttttgtatgc-3' |
| 3' primer for ATF6β CKO 3'-arm | 5'-cccaagcttgaattcccaccctagtcccactaagtgcttaacc-3' |
| 5' primer for ATF6α CKO 5'-pro | 5'-cggggtaccctgactcaagcctcaaaagg-3' |
| 3' primer for ATF6α CKO 5'-pro | 5'-cccaagctttattcagtagccccactagg-3' |
| 5' primer for ATF6β CKO 5'-pro | 5'-cggggtaccaagactgacagctgtgtgtctacagg-3' |
| 3' primer for ATF6β CKO 5'-pro | 5'-cccaagcttgctgagaccatgtcttgcctgtgccaaacc-3' |
