# Supplementary material for: Partial limitation of cellular functions and compensatory modulation of unfolded protein response pathways caused by double-knockout of ATF6α and ATF6β
Source: Cell Stress Chaperones. 2023 Nov 20;29(1):34–48. doi: 10.1016/j.cstres.2023.11.002 (PMC10939067; doi:10.1016/j.cstres.2023.11.002)
Supplement: Supplementary file 3 — Supplementary material [file mmc3.docx]

|  | **Table S2. Akai R. et al.** |
| --- | --- |
| Table S2. Information on primers for genotyping PCR of *ATF6a* conditional KO mice and *ATF6β* conditional KO mice | |
| Application | Sequence |
|  | 5'-tgcatacctggcactgttcg-3' |
| *ATF6α* conditional KO mice | 5'-aaaggtgtgagccctagtgc-3' |
|  | 5'-tccggtgtacatgtaagtcc-3' |
|  | 5'-agtggatgtatgtgccaagg-3' |
| *ATF6β* conditional KO mice | 5'-ggtagcatggacagcaatgg-3' |
|  | 5'-gcctgtgagccacttacacg-3' |
