# Supplementary material for: Partial limitation of cellular functions and compensatory modulation of unfolded protein response pathways caused by double-knockout of ATF6α and ATF6β
Source: Cell Stress Chaperones. 2023 Nov 20;29(1):34–48. doi: 10.1016/j.cstres.2023.11.002 (PMC10939067; doi:10.1016/j.cstres.2023.11.002)
Supplement: Supplementary file 4 — Supplementary material [file mmc4.docx]

|  | | **Table S3. Akai R. et al.** | |
| --- | --- | --- | --- |
| Table S3. Information on primers for RT-PCR analyses of gene expression | | | |
| Application | 5' primer | | 3' primer |
| *ATF6α* | 5'-gcagcggatgataaagaacc-3' | | 5'-tcatgggcccatagttcagc-3' |
| *ATF6β* | 5'-cagcggatgatcaagaatcg-3' | | 5'-cacaggcccaaagttgaagg-3' |
| *BiP* | 5'-atgatgaagttcactgtggtgg-3' | | 5'-cacccaggtcaaacacaagg-3' |
| *XBP1* | 5'-gaaccaggagttaagaacacg-3' | | 5'-aggcaacagtgtcagagtcc-3' |
| *GADD34* | 5'-ctgatgggcttgctcagtcg-3' | | 5'-gcctcttatcagccccttgc-3' |
| *CReP* | 5'-ccggaaatccaccaccttcg-3' | | 5'-agccatcgtcctcaggttcc-3' |
| *GAPDH* | 5'-ctgaacgggaagctcactgg-3' | | 5'-caccaccctgttgctgtagc-3' |
