# Supplementary figures and images for: Partial limitation of cellular functions and compensatory modulation of unfolded protein response pathways caused by double-knockout of ATF6α and ATF6β
Source: Cell Stress Chaperones. 2023 Nov 20;29(1):34–48. doi: 10.1016/j.cstres.2023.11.002 (PMC10939067; doi:10.1016/j.cstres.2023.11.002)

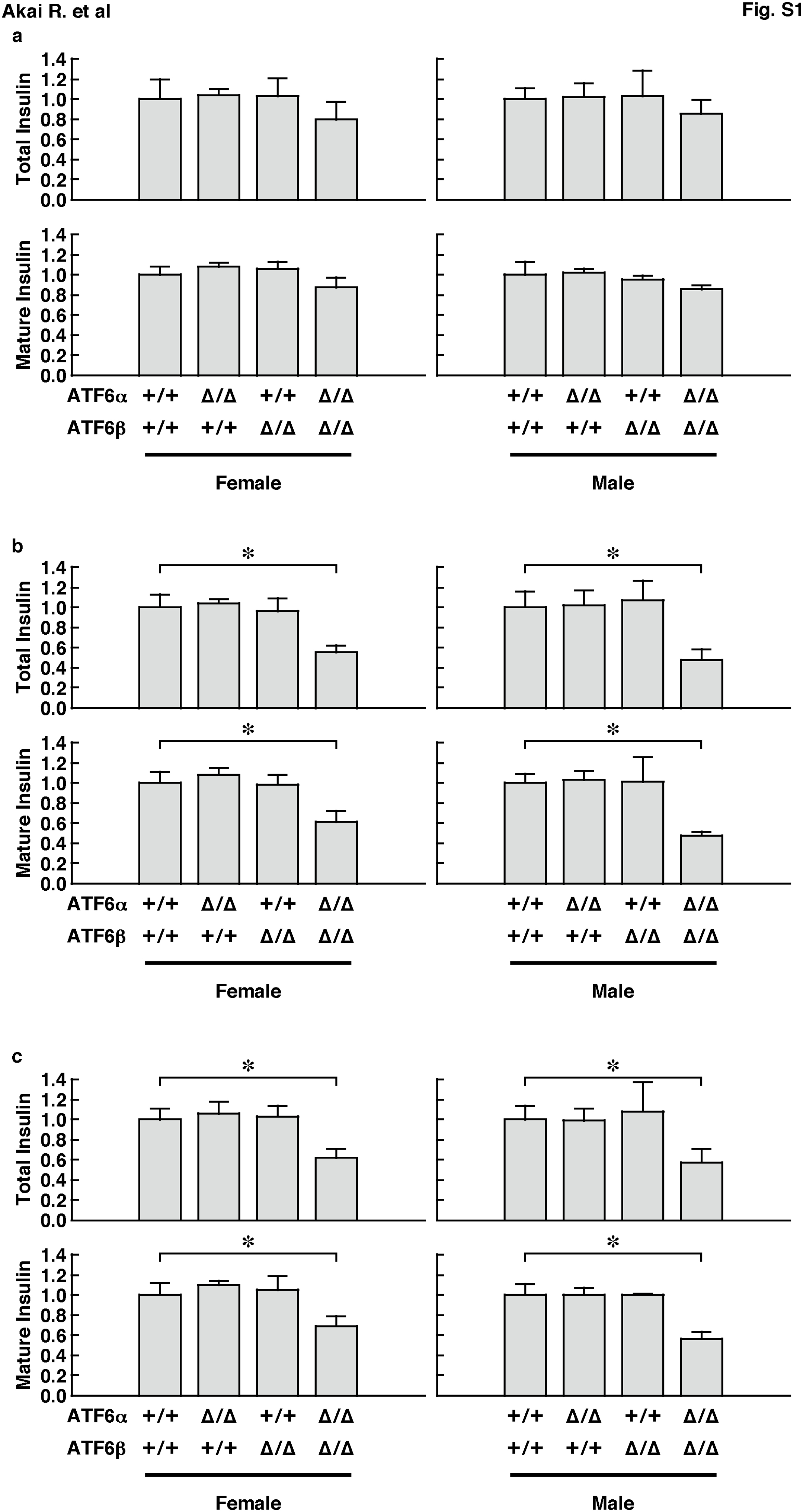

Supplement: Supplementary file 5 — Supplementary material [file mmc5.jpg]

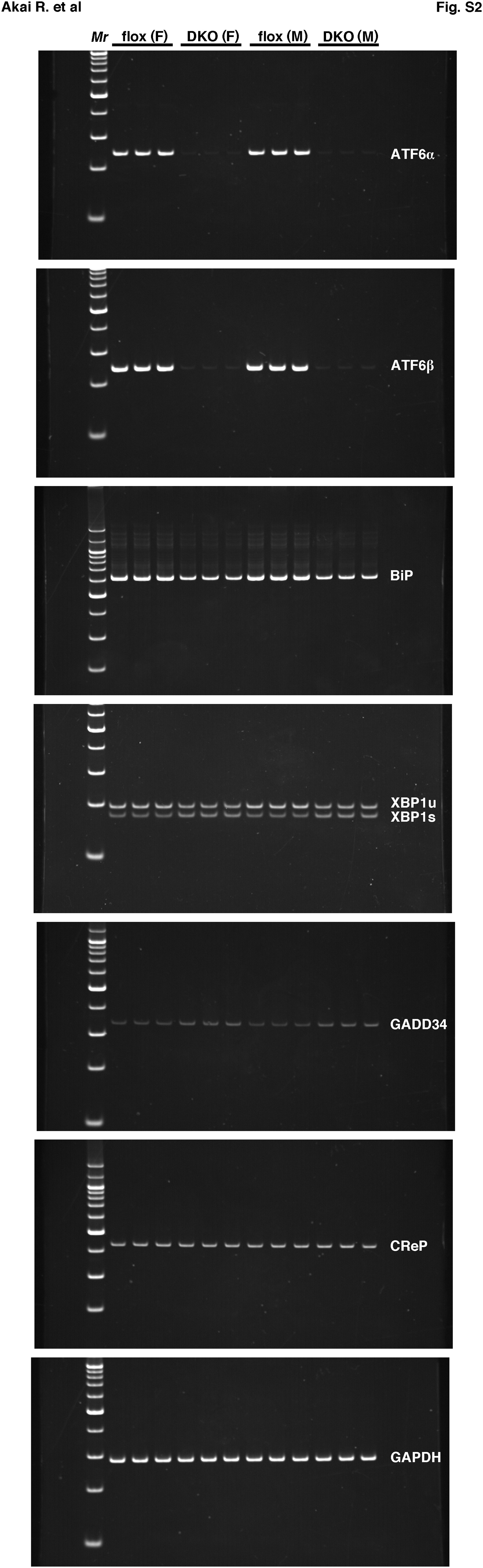

Supplement: Supplementary file 6 — Supplementary material [file mmc6.jpg]

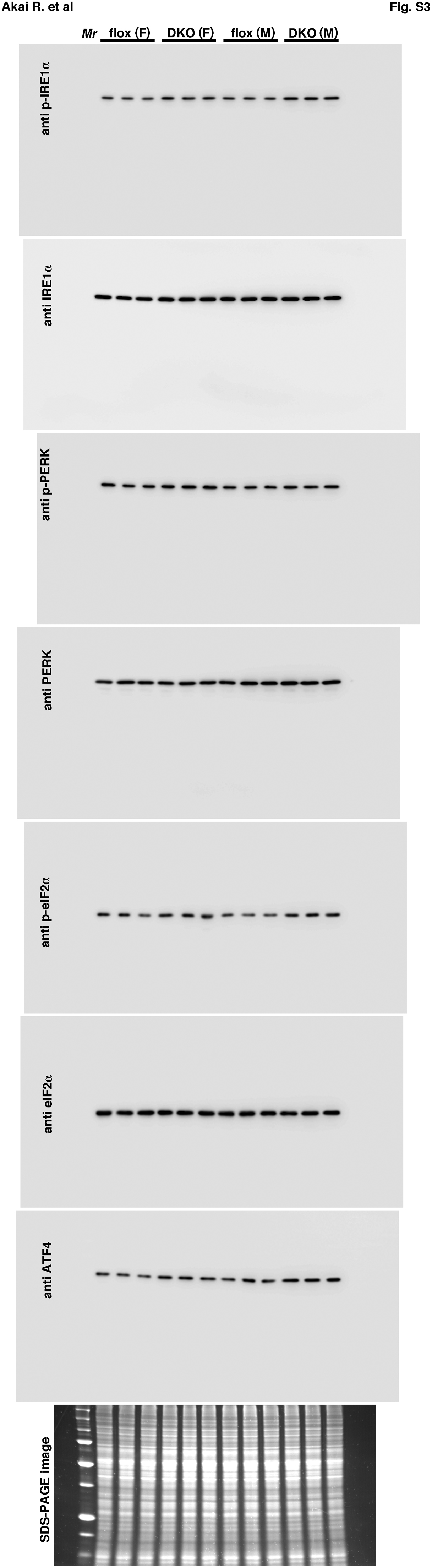

Supplement: Supplementary file 7 — Supplementary material [file mmc7.jpg]
